# Nuclear Accumulation of HSP70 in Mouse Skeletal Muscles in Response to Heat Stress, Aging, and Unloading With or Without Reloading

**DOI:** 10.3389/fgene.2018.00617

**Published:** 2018-12-20

**Authors:** Antonios Apostolopoulos, Ayane Nakamura, Shingo Yokoyama, Megumi Aoshima, Risa Fujimoto, Kodai Nakamura, Rika Ito, Katsumasa Goto

**Affiliations:** ^1^ Centre of Human and Applied Physiological Sciences, School of Basic and Medical Biosciences, King’s College London, London, United Kingdom; ^2^ Department of Physiology, Graduate School of Health Sciences, Toyohashi SOZO University, Toyohashi, Japan; ^3^ Biological Sciences, Graduate School of Sciences and Technology for Innovation, Yamaguchi University, Yamaguchi, Japan

**Keywords:** skeletal muscle, HSP70, Hikeshi, aging, unloading, reloading, heat stress

## Abstract

The purpose of this study was to investigate the nuclear accumulation of heat shock protein 70 (HSP70), a molecular chaperonin in mouse skeletal muscle in response to aging, heat stress, and hindlimb unloading with or without reloading. Profiles of HSP70-specific nuclear transporter Hikeshi in skeletal muscles were also evaluated. Heat stress-associated nuclear accumulation of HSP70 was observed in slow soleus (SOL) and fast plantaris (PLA) muscles of young (10-week-old) mice. Mean nuclear expression level of HSP70 in slow medial gastrocnemius (MGAS) and PLA muscles of aged (100-week-old) mice increased ~4.8 and ~1.7 times, compared to that of young (10-week-old) mice. Reloading following 2-week hindlimb unloading caused accumulation of HSP70 in myonuclei in MGAS and PLA of young mice ( *p* < 0.05). However, reloading-associated nuclear accumulation of HSP70 was not observed in both types of muscles of aged mice. On the other hand, 2-week hindlimb unloading had no impact on the nuclear accumulation of HSP70 in both muscles of young and aged mice. Nuclear expression level of Hikeshi in both MGAS and PLA in mice was suppressed by aging. No significant changes in the nuclear Hikeshi in both muscles were induced by unloading with or without reloading. Results of this study indicate that the nuclear accumulation of HSP70 might show a protective response against cellular stresses in skeletal muscle and that the protective response may be suppressed by aging. Protective response to aging might depend on muscle fiber types.

## Introduction

Skeletal muscle has a great potential in response to various environmental stimuli. Mechanical loading is one of the most important factors that regulates skeletal muscle mass. Increase in mechanical load on skeletal muscle results in hypertrophy, whereas unloading such as in spaceflight or hindlimb suspension results in atrophy (Hauschka et al., 1987; Martin et al., 1988; Ohira, 2000; Naito et al., 2000; Goto et al., 2003; Sandona et al., 2012). Additionally, aging also causes decrease in skeletal muscle function and mass, so-called sarcopenia (Rosenberg, 1997; Frontera et al., 2000), which is dominant in fast muscle (Evans and Cambell, 1993). Therefore, the molecular mechanism of sarcopenia might be different from that of unloading-associated muscle atrophy, which is dominant in slow skeletal muscle, and a slow-to-fast transition shift of myosin phenotypes is observed in unloading-associated atrophied muscle (Templeton et al., 1984; Ohira et al., 1992). However, the reason why aging-associated muscle atrophy is dominant in fast muscle remains unclear.

Oxidative stress is considered to be a cause of skeletal muscle atrophy (Enoki et al., 2016). In fact, antioxidants such as catechin and astaxanthin show indications that they can attenuate unloading-associated skeletal muscle atrophy (Cremades et al., 2007; Ota et al., 2011). Additionally, long-term catechin ingestion with heat stress application might improve impaired function of elderly women (Goto et al., 2014). Therefore, oxidative stress may also play a role in aging-associated skeletal muscle atrophy (Carter et al., 2007). However, aging- and unloading-associated cellular stress in skeletal muscle cell remains unclear.

Various environmental stresses upregulate heat shock proteins (HSPs), especially 70 kDa HSP (HSP70), *via* the stress response (Schlesinger, 1990; Charveron et al., 1995; Minowada and Welch, 1995; Matz et al., 1995; Laplante et al., 1998; Cao et al., 1999; Latchman, 2001). HSP70 including inducible 70 kDa HSP (so-called HSP72) acts as a molecular chaperone and plays a part in the tightly regulated systems for the maintenance of cellular homeostasis (Welch, 1992; Latchman, 2001). Heat shock-induced nuclear accumulation of HSP70 has been well observed (Pelham, 1984; Velazquez and Lindquist, 1984; Welch and Feramisco, 1984; Lamian et al., 1996). Nuclear HSP70 has been proposed to exhibit a protective role for various gene expressions in the nucleus against cellular stresses by protecting the quality and integrity of DNA (Kotoglou et al., 2009).

HSP70 is also upregulated in response to the factors that can affect muscle plasticity (Koya et al., 2013; Ohno et al., 2015; Yokoyama et al., 2016). It has been shown that the upregulation of HSP70 in response to heat stress prevents disuse or immobilization-induced muscle atrophy by having anti-apoptotic effects on skeletal muscle (Naito et al., 2000; Senf et al., 2008). Upregulation of HSP70 in skeletal muscle is also observed during muscle hypertrophy (Goto et al., 2002; Koya et al., 2013) and regrowth of unloading-associated atrophied muscle (Yasuhara et al., 2011). Additionally, reports show an upregulation of HSP70 with aging in the skeletal muscle of rodents (Chung and Ng, 2006) as well as humans (Beltran Valls et al., 2015). These reports show that HSP70 might play an important role in the preservation of skeletal muscle mass, even though the exact role of HSP70 in skeletal muscle cells has yet to be elucidated. However, all these studies concern the cytoplasmic fraction of HSP70, while there is no report for the nuclear expression level of HSP70 in skeletal muscle.

Recently, Hikeshi, a novel protein that is encoded by chromosome 11 open reading frame 73 (the C11orf73; Gene ID: 51501), was identified and proposed to be the nuclear import carrier of HSP70 under stress (Kose et al., 2012). However, few studies have been conducted concerning Hikeshi, and there has been no evidence regarding its expression levels and its physiological role in skeletal muscle.

In this study, therefore, we investigated the nuclear accumulation of HSP70 in mouse skeletal muscle in response to aging, heat stress, and hindlimb unloading with or without reloading. Profiles of Hikeshi in skeletal muscles were also evaluated.

## Materials and Methods

### Animals

Young (10-week-old) and aged (100-week-old) male C57BL/6J mice were used in this study. All animal protocols were conducted in accordance with the Japanese American Physiological Society Guide for the Care and Use of the Laboratory Animals, as adopted and promulgated by the National Institutes of Health (Bethesda MD), and were approved by the Animal Use Committee of Toyohashi SOZO University (A2014002, A2015002, and A20166004). All efforts were made to prevent discomfort and suffering. Three to five mice were housed in a home cage (20 × 31 cm and 13.5 cm height) in a clean room with controlled temperature and humidity at approximately 23°C and 55%, respectively, with a 12/12 h light-dark cycle. Solid diet and water were provided *ad libitum.*

### Experiment 1

We investigated the effects of heat stress on the nuclear accumulation of HSP70 in slow soleus (SOL) and fast PLA muscles. Male C57BL/6J mice (10-week old, *n* = 6) were randomly divided into control and heat-stressed groups (*n* = 3 in each group).

#### Exposure to Heat Stress

Mice in the heat-stressed group were exposed to heat stress (41°C) for 60 min in a heating chamber without anesthesia. Heating protocol in the present study caused an increase in colonic temperature up to 41°C and induced the upregulation of heat shock proteins in mammalian skeletal muscle (Kobayashi et al., 2005; Kojima et al., 2007). Approximately 30–45 min after the beginning of heat stress, the colonic temperature of the mice increased up to 41°C. At the end of heating, the colonic temperature ranged from 41 to 42°C. Therefore, the core temperature of mice was maintained at ~41°C for at least 15 min. During heating, mice freely had access to diet and water in the cage. Following heating, all mice were housed in a room, which maintained a constant temperature of ~23°C. No abnormalities in the moving of mice were observed, and no mice died during and after the heating.

#### Sampling

Mice from the heat-stressed group were culled 24 h after the application of heat stress. Soleus (SOL) and plantaris (PLA) muscles were dissected from both hindlimbs, trimmed of excess fat and connective tissues, and weighted. They were then frozen in liquid nitrogen and stored at −80°C. Dissection of the same muscles of the control group was also carried out on the same day following the same procedure. The nuclear accumulation of HSP70 in SOL and PLA muscles was evaluated by immunohistochemical and immunoblotting analyses.

### Experiment 2

We also investigated the nuclear expression level of HSP70 and Hikeshi proteins in slow SOL and fast PLA muscles of young and aged mice in response to unloading with or without reloading. Young (10-week old, *n* = 18) and aged (100-week old, *n* = 18) C57BL/6J male mice were used. Mice of both aged groups were randomly divided into the untreated pre-experimental control (*n* = 6 in each group) and the hindlimb-suspended groups (*n* = 12 in each group).

#### Hindlimb Suspension

Mice from hindlimb-suspended groups were subjected to continuous hindlimb suspension for 2 weeks. Hindlimb suspension was performed following the same methods as described previously (Yasuhara et al., 2011). Briefly, tails of the mice were cleaned and were loosely surrounded by adhesive tapes cross sectionally, with a string fixed at the dorsal side of the tail, to keep the blood flow intact. The string was fastened to the roof of the cage at a height, which allowed the forelimbs to support their weight, yet preventing the hindlimbs from touching the floor and the sides of the cage (20 × 31 cm and 13.5 cm height). During the 2-week suspension, mice could reach food and water freely by using their forelimbs. Immediately after the 2-week hindlimb suspension, ambulation recovery was allowed for some mice in the suspended group (*n* = 6 of each age group). During the recovery, mice were housed in cages of the same size as described previously. Mice in the pre-experimental control group were also housed in a cage of the same size.

#### Sampling

Slow medial gastrocnemius (MGAS) and fast PLA muscles were dissected at baseline (untreated pre-experimental control; Pre), at 0 (*R0*) and 2 weeks (*R0*) after 2-week hindlimb suspension (*R2*). In this experiment, soleus muscle was not used, since soleus muscle is very small, making it difficult to prepare the nuclear fraction of muscle proteins. Dissected muscles were trimmed of excess fat and connective tissues, weighed, frozen in liquid nitrogen, and stored at −80°C until analyzed. The nuclear expression level of HSP70 and Hikeshi proteins in MGAS and PLA muscles was evaluated by immunoblotting analyses.

#### Immunohistochemical Analyses

Serial transverse cryosections (7-μm thick) of the midbelly region of the frozen soleus and plantaris muscles were cut at −20°C and mounted on slide glasses. The sections were air dried and stained to analyze the translocation of HSP72 into the nucleus following a standard immunohistochemical technique. Cross sections were fixed with paraformaldehyde (4%) and then were post-fixed in ice-cold methanol. After blocking by using a reagent (1% Roche Blocking Reagent, Roche Diagnostics, Penzberg, Germany), samples were incubated with primary antibodies for HSP70 (diluted 1:200; ab79852, Abcam, Cambridge, UK). Sections were also incubated with secondary antibodies for Cy3-conjugated anti-rabbit immunoglobulin G (IgG) (diluted 1:200; Jackson Immuno Research, West Grove, PA, USA) for 1 h at room temperature. Nuclei were then stained in a solution of 4′,6-diamidino-2-phenylindole dihydrochloride (Dapi, 1 μg/ml; Sigma-Aldrich, St. Louis, MO, USA) for 15 min at room temperature. The images of muscle sections were incorporated into a personal computer (Keyence BZ-X viewer) by using a microscope (BZ-X700, Keyence, Osaka, Japan).

#### Immunoblotting Analyses

The expression levels of HSP70 and Hikeshi proteins were assessed by a standard immunoblotting assay, as described previously (Yasuhara et al., 2011; Koya et al., 2013; Nishizawa et al., 2013). Frozen SOL, MGAS, and PLA were homogenized in STM buffer [1 M sucrose, 1 M Tris, pH 7.4, 1 M MgCl_2_, 1% protease/phosphatase inhibitor cocktail (Cell Signaling Technology, Danvers, MA, USA)] with a glass homogenizer. The homogenates were centrifuged at 800 × *g* (4°C for 15 min), and the supernatant was aspirated. The pellet was re-suspended with STM buffer, sonicated, and centrifuged at 800 × *g* (4°C for 15 min). The supernatant was also aspirated. The pellet was re-suspended in NET buffer (Cell Signaling Technology), sonicated, and centrifuged at 11,000 × g (4°C for 15 min), and the supernatant was collected as the nuclear fraction.

The concentration of proteins in the nuclear fraction was evaluated using CB-X^TM^ Protein assay kit (G-Bioscience, St Louis, MO, USA). A part of the supernatant was solubilized in SDS sample buffer at a constant concentration of protein and was incubated at 95°C for 5 min. SDS-polyacrylamide gel electrophoresis (PAGE) was carried out on 14% polyacrylamide containing 0.5% SDS at a constant current of 20 mA for 120 min (Bio-Rad PowerPac Universal, Hercules, CA, USA). Equal amounts of protein were loaded on each gel. Molecular weight markers (#161-0374, Bio-Rad, Hercules, CA, USA) were applied to both sides of 24 lanes as the internal controls for the transfer process and electrophoresis.

Following SDS-PAGE, proteins were transferred to polyvinylidene difluoride (PVDF) membranes (0.2-μm pore size, Bio-Rad, Hercules, CA, USA) at a constant voltage of 100 V for 60 min at 4°C. The membranes were blocked for 1 h at room temperature in a blocking buffer: 5% (w/v) skim milk with 0.1% Tween 20 in Tris-buffered saline (TBS) at pH 7.5. The membranes were then incubated for 2 h with polyclonal antibodies for HSP70 (diluted 1:10,000; SPA-812, Stressgen, Victoria, BC, Canada; 1:10,000; ab79852, Abcam), and Hikeshi (diluted 1:2,000; 14808-1-AP, Proteintech, IL, USA) at room temperature. Membranes were thereafter reacted with secondary antibodies for 1 h (goat anti-rabbit IgG horseradish peroxidase-linked antibody, Cell Signaling Technology). After the final wash, protein bands were visualized by chemiluminescence (ImmunoStar LD, Wako Pure Chemical Industries, Osaka, Japan), and signal density was measured by the ChemiDoc Touch MP Imaging system (Image Lab Software version 5.2.1, Bio-Rad, Hercules, CA, USA) or ImageJ software (National Institute of Health, MD, USA). Each sample was investigated in duplicate, at least, to ensure that the results were not influenced by loading errors. Ponceau staining was carried out as the internal controls for the transfer process and electrophoresis. Total protein was stained by Ponceau in each lane. Cumulative signal density of all stained bands was scanned and was calculated as total protein in each lane. All blotting data were normalized with total protein in each sample. Protein expression level of HSP70 and Hikeshi is reported as the relative value to the control group or the expression level (1.0) of 10-week-old mice before hindlimb suspension (Pre).

#### Statistical Analysis

All values were expressed as means ± standard error of the mean (*SEM*). Statistical significance was determined by two-way (age × time) analysis of variance (ANOVA) followed by Tukey’s *post hoc* test. The significance level was accepted at *p* < 0.05.

## Results

### Experiment 1

**Figure 1:**
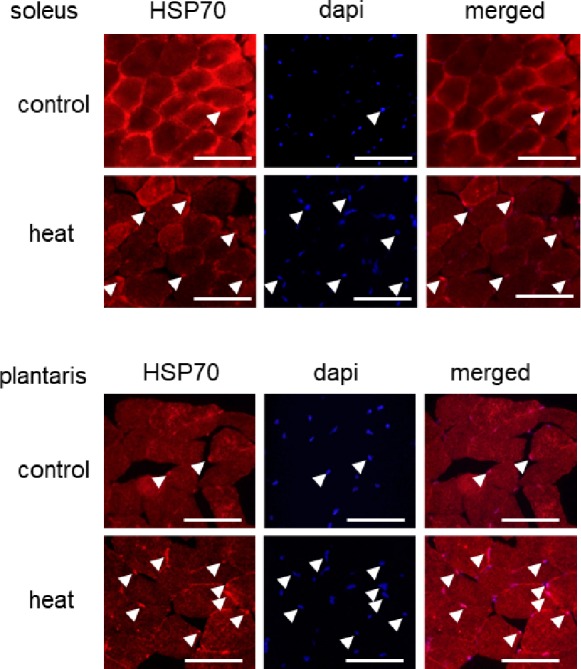
Effects of heat stress on the nuclear accumulation of heat shock protein 70 (HSP70) in mouse skeletal muscle. Arrow head indicates the nuclear accumulation of HSP70. Control: untreated control group, heat: heat-stressed group. Bar: 50 μm.

At first, we investigated the effects of 1-h heat stress on the nuclear accumulation of HSP70 in slow SOL and fast PLA muscles. In this study, there was no significant difference in body as well as in muscle wet weights of the mice between control and heat group, before or after heat stress (data not shown). Figure 1 shows the representative cross-sectional images of slow SOL and fast PLA muscles. HSP70 is expressed in the cytoplasm of both SOL and PLA muscles in the control group. The signal intensity of HSP70 in the cytoplasm in SOL showed to be higher than PLA in the untreated control group.

**Figure 2:**
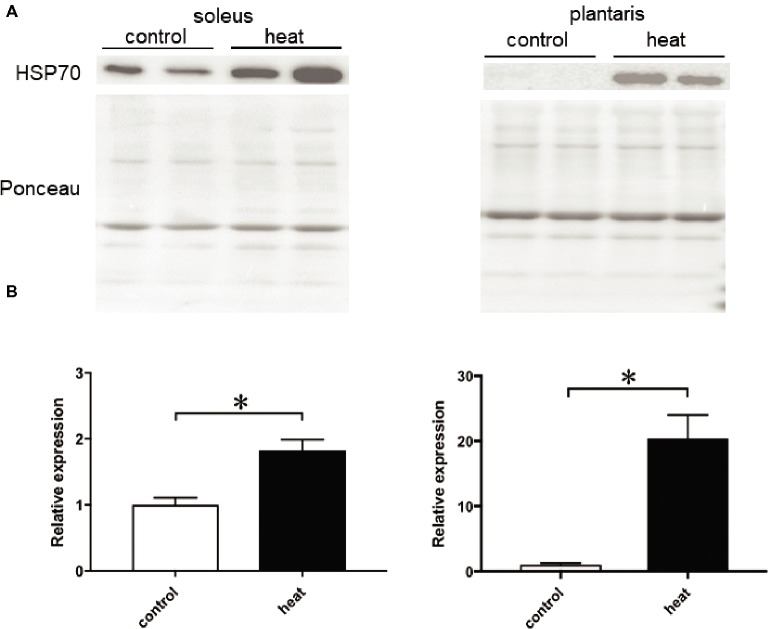
Expression level of heat shock protein 70 (HSP70) in the nuclear fraction of soleus and plantaris muscles in response to heat stress. Representative expression patterns of HSP70 and Ponceau staining in the nuclear fraction **(A)**. Changes in the mean expression levels of HSP70 **(B)**. Control: untreated control group (*n* = 3), heat: heat-stressed group (*n* = 3). The expression level of HSP70 is reported as the relative value to the control group (1.0). Values are expressed as means ± *SEM*. *p < 0.05.

Immunohistochemical analyses demonstrated that HSP70-positive nuclei are more prominent in both SOL and PLA muscles in the heat-stressed group than the untreated control group. Heat stress-associated increase in HSP70-positive nuclei was predominant in slow SOL muscle, compared with fast PLA muscle. Figure 2 shows the mean expression level of HSP70 in the nuclear fraction in both SOL and PLA muscles. Even though a difference in the sensitivity of the nuclear accumulation of HSP70 in response to heat stress between slow SOL and fast PLA muscles has been observed, heat stress induces the nuclear accumulation of HSP70 in skeletal muscle (Figure 2, *p* < 0.05).

### Experiment 2

After the confirmation of heat stress-associated accumulation of HSP70 in skeletal muscles, we also investigated the effects of unloading followed by reloading on the nuclear expression level of HSP70 and Hikeshi proteins in slow MGAS and fast PLA muscles of young and aged mice.

**Figure 3:**
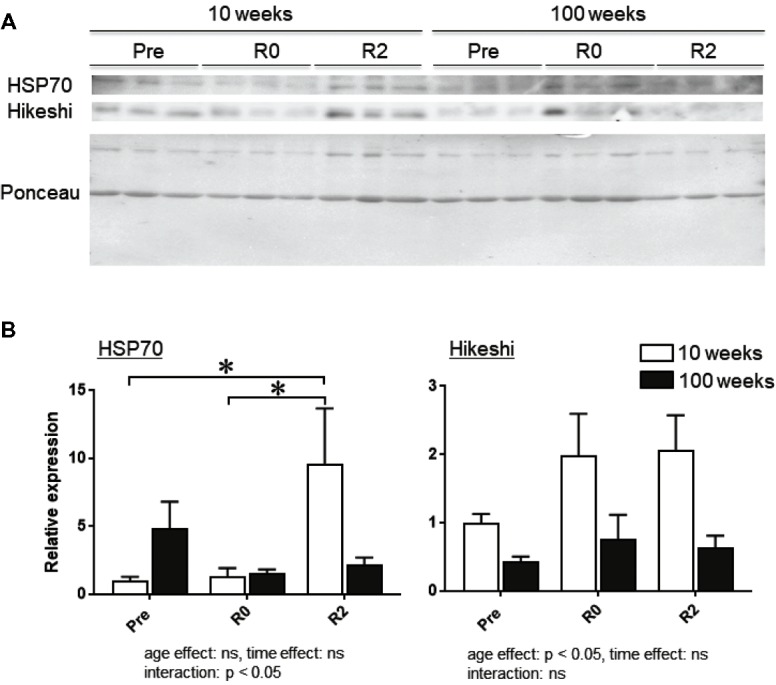
Expression level of heat shock protein 70 (HSP70) and Hikeshi in the nuclear fraction of medial gastrocnemius (MGAS) muscles in young (10-week-old) and aged (100-week-old) mice in response to 2-week hindlimb suspension followed by 2-week ambulation recovery. Representative expression patterns of HSP70 and Ponceau staining in the nuclear fraction **(A)**. Changes in the mean expression levels of HSP70 and Hikeshi **(B)**. 10 weeks: 10-week old, 100 weeks: 100-week old, Pre: before hindlimb suspension, *R0* and *R2*: recovery 0 and 2 weeks, respectively. *n* = 6/each age group at each time point. The expression level of HSP70 and Hikeshi is reported as the relative value to the expression level (1.0) of 10-week-old mice before hindlimb suspension (Pre). Values are expressed as means ± *SEM*. *p < 0.05.

Figure 3A shows the representative expression patterns of HSP70, Hikeshi, and total proteins in the nuclear fraction in MGAS muscle in young and aged mice in response to 2-week-continuous hindlimb unloading with or without 2-week recovery. Two-way ANOVA revealed that there was a significant interaction (*p* < 0.05) in the nuclear expression level of HSP70 (Figure 3B) and a significant main effect of age in the nuclear expression level of Hikeshi (Figure 3C). Mean nuclear expression level of HSP70 in slow medial gastrocnemius (MGAS) of aged (100-week-old) mice increased ~4.8 times, compared to that of young (10-week-old) mice. There was a significant difference in the nuclear expression level of HSP70 between the control (Pre) and 2 weeks after hindlimb suspension (*R2*, *p* < 0.05), immediately after the 2-week hindlimb suspension (*R0*) and *R2* for MGAS muscle (*p* < 0.05) in young mice. No significant effects of hindlimb unloading with or without reloading on nuclear HPS70 in aged mice were reported at any time point in MGAS muscle.

**Figure 4:**
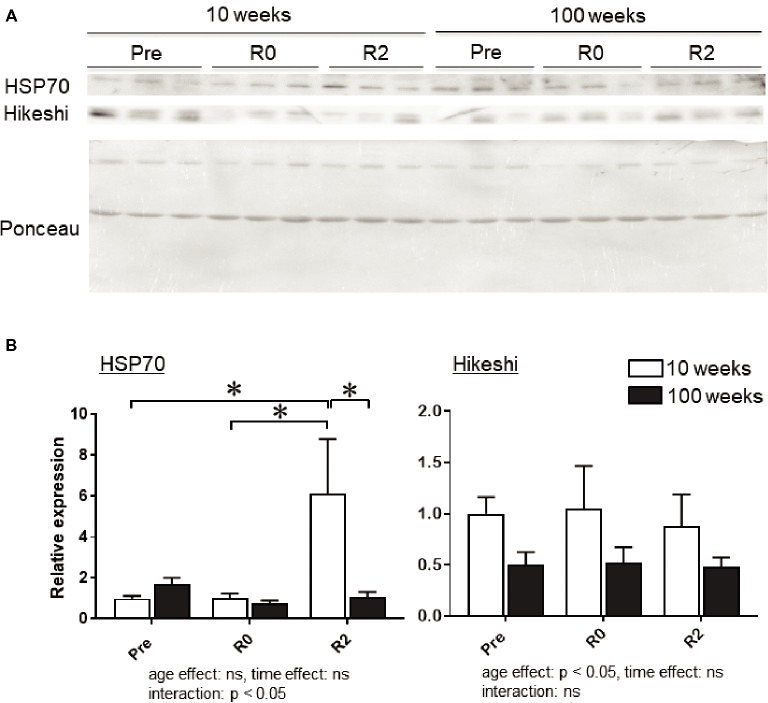
Expression level of heat shock protein 70 (HSP70) and Hikeshi in the nuclear fraction of plantaris (PLA) muscles in young (10-week-old) and aged (100-week-old) mice in response to 2-week hindlimb suspension followed by 2-week ambulation recovery. Representative expression patterns of HSP70 and Ponceau staining in the nuclear fraction **(A)**. Changes in the mean expression levels of HSP70 and Hikeshi **(B)**. Abbreviations are the same as in Figure 2. ns: not significant. *n* = 6/each age group at each time point. The expression level of heat shock protein 70 (HSP70) and Hikeshi is reported as the relative value to the expression level (1.0) of 10-week-old mice before hindlimb suspension (Pre). Values are expressed as means ± *SEM*. *p < 0.05.

Figure 4A shows the representative expression patterns of HSP70, Hikeshi, and total proteins in the nuclear fraction in PLA muscle in young and aged mice in response to 2-week continuous hindlimb unloading with or without 2-week recovery. Two-way ANOVA revealed that there was a significant interaction (*p* < 0.05) in the nuclear expression level of HSP70 (Figure 4B) and a significant main effect of age in the nuclear expression level of Hikeshi (Figure 4C). Mean nuclear expression level of HSP70 in PLA of aged mice increased ~1.7 times, compared to that of young mice. Reloading-associated nuclear accumulation of HSP70 in PLA muscle of young mice was observed, compared to the expression level at basal (Pre, *p* < 0.05) and immediately after unloading (*R0*, *p* < 0.05). Hindlimb unloading with or without reloading had no impact on the nuclear expression of HPS70 in PLA muscle of aged mice. Nuclear expression level of HSP70 in PLA muscle of young mice was significantly higher than aged mice (*p* < 0.05).

## Discussion

The present study demonstrated heat stress-associated nuclear accumulation of HSP70 in slow SOL and fast PLA muscles. Nuclear expression level of HSP70 in slow MGAS and PLA muscles of aged mice increased ~4.8 and ~1.7 times, compared to that of young mice. In young mice, reloading-associated upregulation of HSP70 was observed in both MGAS and PLA muscles, but not in aged mice. No effect of unloading on nuclear HSP70 was observed in both muscles of young and aged mice. No effect of unloading on nuclear HSP70 was observed in both muscles of young and aged mice. Nuclear expression of Hikeshi, a nuclear transporter for HSP70, in MGAS and PLA muscles of aged mice was lower than young mice.

### Heat Stress-Associated Nuclear Accumulation of HSP70

In the present study, heat stress-associated nuclear accumulation of HSP70 was observed in mouse skeletal muscles using a standard immunohistochemical technique. Although many papers report that HPS70 in skeletal muscle is upregulated by heat stress (Goto et al., 2004; Kojima et al., 2007; Ohno et al., 2012), this is the first report showing the nuclear accumulation of HSP70 in response to heat stress. Furthermore, this accumulation was observed in both slow SOL and fast PLA muscles. The results from this study suggest that the nuclear accumulation of HSP70 in response to heat stress appears in the mammalian skeletal muscles regardless of the muscle type. Since in this study, the nuclear accumulation of HSP70 was evaluated by immunohistochemical staining, there was no comparison of HSP70 expression level between nuclear and cytoplasmic fractions. Therefore, it remains unclear whether a nuclear translocation of HSP70 in response to heat stress happens. Even though heating-associated nuclear accumulation of HSP70 in other cells plays a protective role against cellular stress, a physiological role of it in skeletal muscles remains unclear. Further studies are needed to elucidate this issue.

### Aging-Associated Nuclear Accumulation of HSP70

There is also no report regarding the effects of aging on the nuclear expression of HSP70 in skeletal muscle. The present study demonstrated aging-associated increase in myonuclear expression level of HSP70 in slow MGAS (~5.8 times) and fast PLA (~1.7 times) in mice. This is the first report to investigate the effects of aging on the nuclear expression levels of HSP70 in skeletal muscle. A previous study showed a significantly higher expression of HSP70 in the crude cellular fraction of slow soleus muscle in aged mice than the young group (Ohno et al., 2012). Additionally, similar phenomena in the crude cellular fraction of red and white gastrocnemius muscle of aged rats were reported (Chung and Ng, 2006).

In the present study, aging-associated increasing rate of nuclear HSP70 is dominant in slow MGAS, compared to fast PLA. The precise mechanism for the enhancement of nuclear accumulation of HSP70 in slow MGAS muscle, compared to fast PLA muscle, induced by aging cannot be explained at present. Aging may induce an increase in cellular stresses in skeletal muscle cells; thus, HSP70 may accumulate in the myonuclei of slow muscle as part of the protective stress response.

Since it is generally accepted that young mice are more active than aged animals, the level of cellular stresses might be higher in skeletal muscle of young animals. On the other hand, there are other systems against oxidative stress, namely superoxide dismutase (SOD), catalase, and so on, in mammalian skeletal muscle. For example, aging is associated with a decrease in antioxidant efficiency and an increase in oxidative stress damage in human skeletal muscle (Bouzid et al., 2014). In this study, we have focused on the nuclear expression level of HSP and HSP70-Hikeshi system in mouse skeletal muscle. Additional investigations including other systems against cellular stresses are needed to elucidate a role of the nuclear HSP expression and HSP70-Hikeshi system in the development of aging-associated muscle atrophy. However, additional experiments should be needed to elucidate this issue.

### Effects of Unloading With or Without Reloading on Nuclear HSP70

In the present study, in young mice, reloading-associated upregulation of HSP70 was observed in both muscles, but not in aged mice. There is no previous report showing the response of nuclear HSP70 in skeletal muscle to unloading with or without reloading. If stress response induces the nuclear accumulation of HSP70, and this nuclear accumulation has a protective role against cellular stresses, reloading on atrophied skeletal muscle might cause to increase cellular stress in young mice, but not in aged mice. No regrowth of unloading-associated MGAS and PLA was observed in aged mice (Nakamura et al., in preparation to submit). These observations suggest that stress response against reloading may be an important factor for the regrowth of atrophied skeletal muscle in response to reloading.

Although aging-associated muscle atrophy is dominant in fast muscle (Evans and Cambell, 1993), the molecular mechanisms for the fiber type-dependent muscle atrophy are still unclear. The present study showed that aging-associated increasing of nuclear HSP70 was dominant in slow MGAS muscle, compared to fast PLA muscle. Furthermore, reloading-associated expression level of nuclear HSP70 in fast PLA of young mice was significantly higher than the aged mice. On the other hand, there was no significant difference in nuclear HSP70 in slow MGAS after reloading between young and aged mice. If the nuclear accumulation of HSP70 has a protective role against cellular stresses in skeletal muscle, slow MGAS muscle might exhibit a protective capacity against aging-associated muscle atrophy. On the other hand, fast PLA muscle might exhibit atrophy, since no nuclear accumulation of HSP70, which may be a protective response, is not induced by aging. Myonuclear accumulation level of HSP70 might show a protective capacity against aging-associated muscle atrophy.

Even though papers show that hindlimb unloading downregulates HSP70 in the crude fraction of skeletal muscle (Lawler et al., 2006; Lawler et al., 2012), there was no evidence regarding the response of nuclear HSP70 in myonuclei to unloading. In the present study, unloading had no impact on nuclear HSP70 in both muscles of young and aged mice. No stress response in skeletal muscle may be induced by unloading.

### Profiles of Nuclear Hikeshi in Skeletal Muscle

In the present study, aging-associated decrease of Hikeshi in the nuclear fraction was observed in both slow MGAS and fast PLA muscles. On the other hand, there was no change in nuclear Hikeshi in response to unloading with or without reloading. Since Hikeshi is a specific nuclear transport protein of HSP70 (Kose et al., 2012), it was hypothesized that its expression level may regulate the capacity of the nuclear translocation of HSP70 in skeletal muscle. However, the nuclear expression level of Hikeshi could not explain the aging-associated nuclear accumulation neither for slow nor for fast skeletal muscles. Evaluation of total and/or cytoplasmic expression level of Hikeshi might be helpful for the elucidation of the mechanisms of this issue.

In conclusion, the present study investigated the nuclear expression level of HSP70 in mouse slow and fast skeletal muscles in response to heat stress, aging, and unloading with or without reloading. Heat stress as well as reloading on unloading-associated atrophied skeletal muscle caused to accumulate HSP70 in myonuclei of young mice. Aging-associated increase of nuclear HSP70 in slow MGAS muscle was observed. It was dominant, compared with fast PLA muscle. Aging attenuated reloading-associated upregulation of HSP70 in both MGAS and PLA muscles. No impact of unloading on nuclear HSP70 was observed in both muscles of young and aged mice. Nuclear expression of Hikeshi in MGAS and PLA muscles was downregulated by aging. Results of this study indicate that the nuclear accumulation of HSP70 might show a protective response against cellular stresses in skeletal muscle and that the protective response may be suppressed by aging. Protective response to aging might depend on muscle fiber types.

## Authors Contributions

KG conceived and designed the experiments. AA, AN, SY, MA, and RI performed the experiments. AA, MA, RI, and KG analyzed the data. AA, AN, MA, RF, KN, and KG contributed the reagents/materials/analysis tools. AA and KG wrote the paper. AN, SY, MA, and RI contributed to animal care. SY, MA, RI, and KG advised for experimental techniques.

### Conflict of Interest Statement

The authors declare that the research was conducted in the absence of any commercial or financial relationships that could be construed as a potential conflict of interest.

The handling editor declared a shared affiliation, though no other collaboration, with one of the authors AA at the time of the review.
